# Body fat percentage assessment by skinfold equation, bioimpedance and densitometry in older adults

**DOI:** 10.1186/s13690-020-00449-4

**Published:** 2020-07-18

**Authors:** Erika Aparecida Silveira, Larissa Silva Barbosa, Ana Paula Santos Rodrigues, Matias Noll, Cesar De Oliveira

**Affiliations:** 1 Faculty of Medicine, Health Science Post-Graduation Program, Universidade Federal de Goiás, Goiânia, Brazil; 2 Instituto Federal Goiano, Goiânia, Brazil; 3 Department of Epidemiology & Public Health, University College London, London, UK

**Keywords:** Body composition, Adiposity, Anthropometry, Aging, Bioelectrical impedance, Dual energy X-ray absorptiometry

## Abstract

**Background:**

Body fat estimation allows measuring changes over time attributed to interventions and treatments in different settings such as hospitals, clinical practice, nursing homes and research. However, only few studies have compared different body fat estimation methods in older adults with inconsistent results. We estimated body fat percentage (%BF) and the level of agreement among dual energy X-ray absorptiometry (DXA), bioelectrical impedance (BIA) and Durnin & Womersley’s skinfold eq. (SF) in older Brazilian adults aged 60 years and older from the Elderly Project Goiânia, Brazil.

**Methods:**

The analytical sample comprised of 132 participants who had DXA data. The level of agreement for the %BF estimated by BIA, SF and DXA i.e. reference method, was examined using Bland and Altman’s and Lin’s plot.

**Results:**

Overall, women had higher body mass index and %BF values measured by all three methods used. BIA and SF equation showed strong concordance to estimate body fat percentage in all participants (CCC = 0.857 and 0.861, respectively) and among women (CCC = 0.788 and 0.726, respectively) when compared to DXA. However, both methods underestimated body fat percentage in women and men with high body fat percentage. A strong level of agreement was observed between DXA and the anthropometric equation developed by Durnin & Womersley in men (CCC = 0.846), while BIA had a moderate concordance (CCC = 0.505) in this group.

**Conclusion:**

The examined methods indicated different body fat estimates. However, the best agreement was observed between DXA and the anthropometric SF equation for men. Future research in older adults should develop new SF equations considering different ethnic groups.

## Background

The prevalence of obesity has considerably increased worldwide over the last few decades and is a growing concern among older adults [1]. Obesity has been linked to hypertension, dyslipidemia, insulin resistance and diabetes mellitus, which can lead to cardiovascular diseases such as coronary heart disease and ischemic stroke [2–4]. The most widely method used to assess the prevalence of obesity status in population studies is the body mass index (BMI) [1]. However, limitations and controversies about the use of BMI have been highlighted especially due to its underestimation of obesity prevalence. Body composition has been considered a better alternative to BMI in older adults due to age-related increases in body fat percentage (%BF) [5, 6]. High body fat is associated with increased mortality [7] and unsuccessful ageing [8]. Therefore, %BF estimation is essential in epidemiological studies and in health services routine instead of relying only on BMI.

Skinfold (SF) measurements allow the assessment of body composition due to the strong relationship between the amount of subcutaneous fat and total BF [9, 10]. SF is a non-invasive method, easy to be measured and has low operating costs [10]. However, there is still a need to evaluate SF equations’ accuracy and agreement to predict %BF in older adults [11, 12].

The development of methods and/or equations for body composition estimation in older adults that can be used in population surveys while accounting for the age-related changes in body composition remains a major challenge [11, 13–15]. Aging affects the subcutaneous and visceral fat distribution. Thus, it is important to use methods such as bioelectrical impedance (BIA) and dual-energy X-ray absorptiometry (DXA) to analyze the whole body. DXA is the reference standard method for body composition estimation, particularly in older adults, as it directly measures muscle mass, adipose tissue and bone density with both good precision and accuracy [6, 16, 17]. However, its high cost and limited device availability make this method unfeasible in population studies and clinical settings.

Body fat estimation is very important later in life [7, 8, 11, 16, 18, 19]. It improves chronic conditions diagnosis and mortality risk assessment. In addition, body fat estimation allows measuring changes over time attributed to interventions and treatments in different settings such as hospitals, clinical practice, nursing homes and research. However, only few studies [11, 20–24] have compared different body fat estimation methods in older adults with inconsistent results. Therefore, we aimed to evaluate the agreement of BIA and SF equation with DXA, as the reference method, to estimate the BF percentage in older Brazilian adults.

## Methods

### Study design and participants

Analyses for this study used data from the Elderly Project Goiânia [2, 18, 25–27], which aims to evaluate the health and nutritional status in older adults aged 60 years and older. It is a cohort study with multi-stage sampling of non-institutionalized older adults in Goiânia city, capital of the Goiás state, Brazil. The initial cohort sample comprised of 418 older adults selected through a probabilistic sampling. For the present study, only a subsample of 132 participants were randomly selected ensuring the same proportion of age distribution, neighborhood and BMI ranges observed in the initial sample, for further assessments including DXA, BIA and skinfold measurement. The number of participants in the subsample was determined based on the Bland and Altman method [10, 28]. We excluded participants who were institutionalized, had incapacitating diseases that did not permit them from leaving their bed or those with partial or total amputation. Individuals with pacemakers or any type of metal adjacent to their body, which was a contra-indication for BIA and DXA examination, and those who were unable to respond to the questionnaire, for reasons such as severe deafness or muteness, were also excluded. A detailed description of the study can be found elsewhere [2, 18, 25–27].

Selected participants were contacted via telephone and were informed about the aim and procedures to be followed during the data collection stage. They were also advised about the preparation required prior to BIA and DXA assessments. All evaluations were performed on the same day during the morning. In order to minimize errors in body composition assessment by the BIA method, participants were given the following instructions: absolute fasting for at least 4 h before the exams; no exercise within the 12 h before the test; urinate 30 min before the test; no consumption of alcohol and foods containing caffeine within 24 h prior to the test and no diuretic use within 24 h prior to the test.

### Study variables

The following anthropometric measures were collected: weight, height, bicipital, tricipital, subscapular and supra-iliac SFs, BIA and DXA. Trained interviewers conducted a standardized and pre-tested survey. SF anatomical points measured were identified by the procedures described by Lohman, Roche and Martorell [29]. In order to improve all anthropometric measures (SF, weight, height), to ensure greater accuracy and validated it, we performed training to standardize the techniques between researcher who collect those data [30].

Weight was measured using the digital Tanita electronic scale that has a capacity of 150 kg and a precision of 100 g. Height was measured using an inelastic and inextensible tape, with a length, width, and precision of 2.00 m, 2 cm and 0.1 cm, respectively, along with a set square. The measurements were conducted according to the techniques described by Gordon et al. [29]. The BMI value was obtained using these measurements. During the anthropometric measures, participants wore only light or intimate clothes, no shoes and no objects in their pockets, on their hands or on their head.

The SF measurements i.e. bicipital, tricipital, subscapular and supra iliac were performed with a Lange adipometer, with a constant pressure of 10 g/mm^2^ on the contact surface and accuracy of 1 mm, with a 0–65 mm scale. The different sites’ measurements were successively performed, and the final values were obtained as the average of three measurements. The SFs were measured according to the recommendations by Harrison et al. [29].

Body density values were calculated using the older adults-specific equation of Durnin and Womersley [10, 31]:

$$ \mathrm{Men}:\mathrm{D}\left(\mathrm{g}/{\mathrm{cm}}^3\right)=1.1765-0.0744\;{\log}_{10}\left(\Sigma 4\mathrm{SF}\right) $$

$$ \mathrm{Women}:\mathrm{D}\left(\mathrm{g}/{\mathrm{cm}}^3\right)=1.1339-0.0645\;{\log}_{10}\left(\Sigma 4\mathrm{SF}\right) $$

The body density equations were converted into fat percentage, by using the Siri equation: %BF = ((4.95/D) – 4.50) × 100, for the purpose of the analysis. Body density conversion is needed as the criterion measure. Siri equation [32] establishes constants of fat mass and fat free mass.

Body fat percentage was also measured using BIA with the Maltron BF906 device, with an impedance of 200–1000 Ω, precision of ±4 Ω, and a frequency of 50 kHz. The measurement was performed with the participant lying in the supine position. An emitter electrode was placed adjacent to the metacarpal-phalangeal joint of the dorsal surface of the right hand, and the other distally of the transverse arch of the upper surface of the right foot. One detector electrode was placed between the radius and ulna distal prominences of the right wrist, whereas the other electrode was placed between the medial and lateral malleoli of the right ankle. Measurement of the %BF by DXA was obtained through a full body scan using the Lunar DPX-MD PLUS device and software version 7.52.002 DPX-L, calibrated daily. Participants lied down on a table in the supine position and remained immobile during the scan. Individuals wore only an apron, and were barefoot, without any earrings, rings, dental prostheses, or other metallic materials.

Database was established using EPIDATA version 3.1 with double input for consistency checking. All the analyses were performed using STATA version 12.0. Shapiro-Wilk test was used to analyze normality of the distribution. Student’s t-test was used to compare %BF average values between men and women. Concordance between the %BF measured by the Durnin and Womersley equation and BIA with the %BF measured by DXA (standard reference), was assessed using the concordance correlation coefficient (CCC) or Lin plots proposed by Bland and Altman [28, 33, 34]. CCC combines precision and accuracy to establish whether the observations deviate significantly from the line of perfect concordance (45°). A value of one corresponds to the regression line lying exactly on the line of perfect concordance [33]. The following cut-off points were adopted: negligible concordance (CCC = 0.00–0.10); weak concordance (CCC = 0.10–0.39); moderate concordance (CCC = 0.40–0.69); strong concordance (CCC = 0.70–0.89) and very strong concordance (CCC = 0.90–1.00) [35].

Bland and Altman’s protocol includes the plotting of a concordance graph (average versus difference), and the calculation of the limit of concordance [28, 34]. This technique allows the visual assessment of the concordance and of the 95% concordance limit.

### Ethics approval and informed consent

This study was conducted according to the guidelines laid down in the Declaration of Helsinki. All participants gave written informed consent. The Research Ethics Committee has approved the Elderly Project Goiânia (number: 031/2007).

## Results

DXA and BIA were performed in 132 participants from the initial cohort. All the variables included in this analysis were normally distributed. Women exhibited higher %BF values in all the methods used (*p* < 0.001) (Table 1).

**Table 1:** Age and different body composition measures distribution by gender

Variables	Male (*n* = 52)		Female (*n* = 80)		Value
	Mean ± SD	Range	Mean ± SD	Range	*p*
Age (years)	70.50 ± 6.68	60.00–91.00	69.69 ± 6.23	60.00–86.00	0.642
BMI (kg/m^2^)^1^	25.75 ± 4.05	14.42–35.36	27.37 ± 5.75	13.67–40.02	0.093
%BF D&W equation^2^	27.88 ± 6.75	8.09–39.68	39.99 ± 5.22	18.38–50.03	0.0001*
%BF BIA^3^	27.90 ± 7.00	3.10–38.90	39.28 ± 8.66	13.80–55.00	0.0001*
%BF DXA^4^	30.21 ± 8.63	5.40–47.40	42.82 ± 9.00	11.20–57.10	0.0001*

^1^Body mass index; ^2^ Body fat percentage estimated with the SF equation of Durnin & Womersley; ^3^ Body fat percentage estimated with bioelectrical impedance (BIA); ^4^ Body fat percentage estimated using dual energy X-ray absorptiometry (DXA); *Significant differences between sexes

**Fig. 1:**
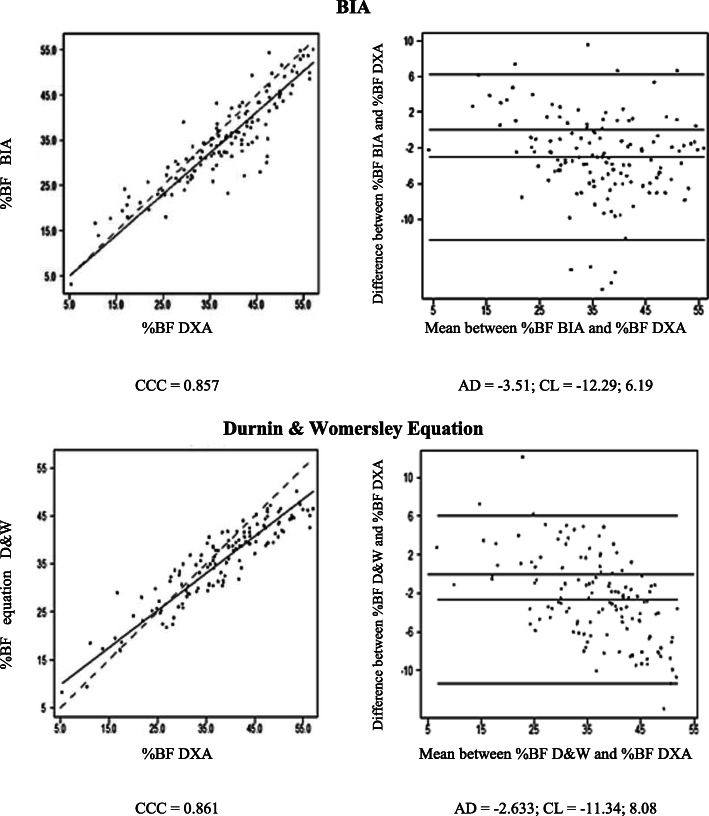
Concordance plots for body fat percentage (%BF) in older adults estimated by dual energy X-ray absorptiometry (DXA), bioelectrical impedance (BIA) and the Durnin & Womersley skinfold eq. (D&W), according to Lin’s concordance correlation coefficient (CCC) and Bland and Altman’s average difference (AD) and 95% concordance limits (CL) for all participants. Continue line = the perfect concordance line, dotted line = the real value in Lin Graph

**Fig. 2:**
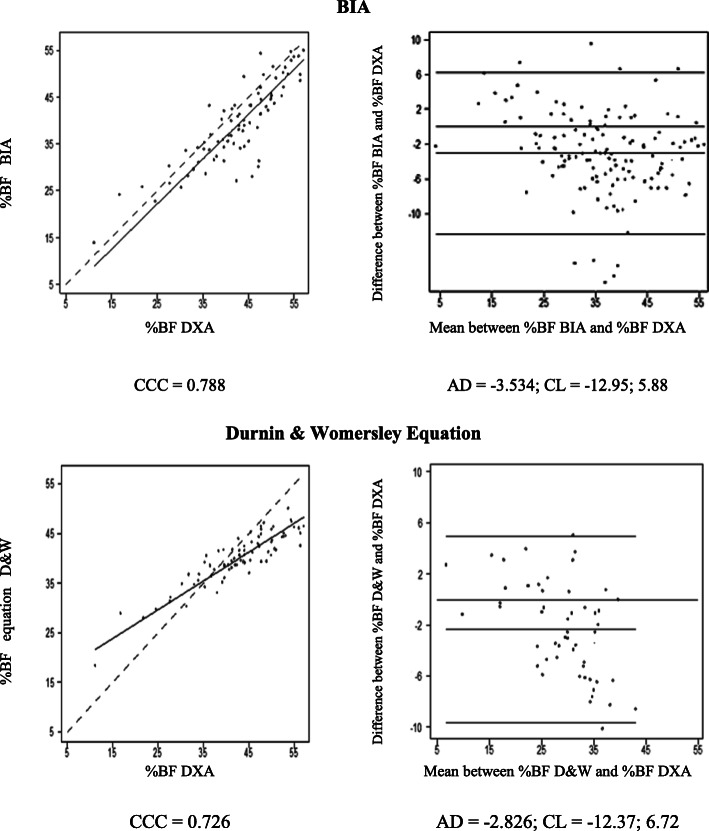
Concordance plots for body fat percentage (%BF) in women estimated by dual energy X-ray absorptiometry (DXA), bioelectrical impedance (BIA), and the Durnin & Womersley equation, according to Lin’s concordance correlation coefficient (CCC) and Bland and Altman’s average difference (AD) and 95% concordance limits (CL). Continue line = the perfect concordance line, dotted line = the real value in Lin Graph

**Fig. 3:**
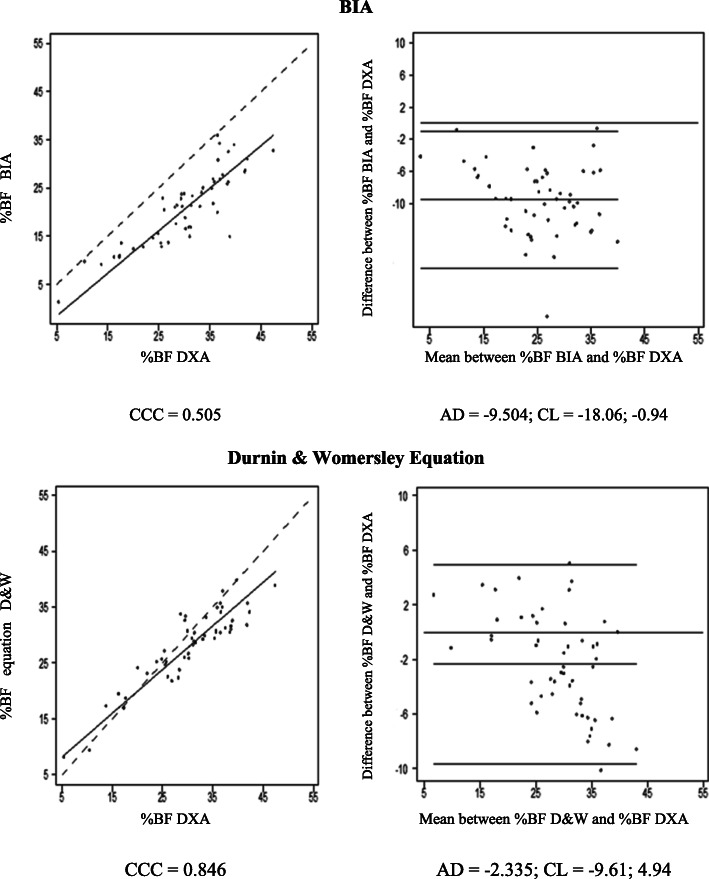
Concordance plots for body fat percentage (%BF) in men estimated by dual energy X-ray absorptiometry (DXA), bioelectrical impedance (BIA), and the Durnin & Womersley equation, according to Lin’s concordance correlation coefficient (CCC) and Bland and Altman’s average difference (AD) and 95% concordance limits (CL). Continue line = the perfect concordance line, dotted line = the real value in Lin Graph

The dispersion of %BF values estimated by BIA and SF equation compared to DXA values are displayed in Figs. 1 to 3. The concordance correlation coefficient analysis showed a strong concordance between BIA and SF equation for all participants and among women (Figs. 1 and 2), whereas a moderate concordance (0.51) was observed for BIA in men (Fig. 3). The higher concordance in our analyses (0.85 - strong concordance) was observed for %BF evaluated by SF equation in men (Fig. 3), followed by BIA in women (0.79 - strong concordance) (Fig. 2).

The analysis of the regression line slope for all participants and between men and women based on the Lin plots showed that an underestimation of %BF by BIA occurs for all levels of BF. However, this underestimation increases at high levels, mainly after 40%, when using SF equation (Figs. 2 and 3).

The graphical approach of Bland and Altman showed that BIA and SF equation tend to underestimate the %BF values in both genders with wide limits of concordance. The underestimation of %BF by BIA in men was 9.5 and 3.5% in women, while for SF equation for both groups was around 2.3 and 2.8%, respectively. Overall, BIA had higher values of underestimation compared to SF equation (Figs. 2 and 3).

## Discussion

In the present study, our main findings showed that values obtained through both methods i.e. BIA and SF equation underestimated %BF in all participants and between men and women. This underestimation increased in the higher values of %BF, mainly after 40% for the SF equation. However, the CCC that measured accuracy and precision between the three methods investigated in the present study was more than 0.85 for all participants. This value decreased in women (0.72) but remained in the strong concordance range. On the other hand, among men, BIA assessment provided the worst level of agreement i.e. moderate (CCC = 0.5), with an underestimated average of 9.5% and had a large limit of agreement. Previous research comparing BIA and SF equations against DXA showed inconsistent findings in older adults related to sex and age-related changes in hydration and bone content and mineralization [15, 21, 24]. When comparing results based on different evaluation methods, it is fundamental to establish whether the measures are either underestimating or overestimating in relation to others [36, 37].

This study represents an important contribution to the literature comparing different methods to estimate %BF in older adults since there are few studies [11, 20] addressing the same aim of our study. At present, there is only another study in older adults [11], aged 65 years and older, and the other studies were conducted in middle-aged adults.

The SF anthropometric equations [31] were based on the calculation of body density, assessed by submerged weight, in women aged > 50 years. The two-compartment model (hydrostatic weighing), which divides the body into fat-free mass and fat mass, was used to develop this equation, and its use has been questioned in older adults. The fat redistribution, loss of compressibility of SFs and the BF and fat free mass constants, adopted by this method, do not reflect the specific characteristics of older adults, which can significantly affect the reliability of BF estimates [38]. Despite the criticism to this method, our results were quite positive and show strong accuracy of SF equation in both genders, mainly in men.

BIA has been recommended as an alternative method to estimate body fat percentage, when DXA cannot be used, because of the high concordance between the two methods in middle-aged adults. This evaluation should be performed in individuals who are within the normal range of total body fat, since BIA tends to overestimate the %BF in lean individuals and to underestimate the %BF in obese individuals [17, 38]. Our results are in agreement with previous studies on middle-aged individuals, wherein BIA underestimated the %BF [17, 39]. The accuracy of the BIA Maltron BF906 equipment used in this study has been previously evaluated in Brazilians against a gold-standard method i.e. hydrostatic weighing showing a moderate concordance correlation (*r* = 0.70 and 0.75, *p* = < 0.01) [40, 41]. The Maltron BF906 was the best BIA equipment compared to other three models analyzed.

Our findings indicated differences in anthropometric and body composition measures between men and women which is consistent with previous studies [42, 43]. The %BF, evaluated via DXA, BIA, and SF equation, in women was higher than that in men, coherent with that noted in previous studies [42, 43]. The SF thickness (subscapular, tricipital, bicipital, and supra-iliac) was also higher in women, which may be related to the larger distribution of subcutaneous fat in women [16]. We highlight that body composition analysis and the accuracy of methods should be stratified by gender especially in older adults.

Durnin and Womersly SF equation adopted in this study is one of the most used and accurate method to evaluate body fat [10–12, 31]. Age, sex and obesity are important factors in terms of SFs and body density measures, since the same SF level at different ages may be associated with changes in the fat distribution pattern [31]. Despite in the present study the CCC for SF equation in both sexes shows strong agreement, it tend to underestimate %BF of those with more than 40% of body fat and the concordance limit in Bland and Altman’s graphic tend to underestimate. A previous study had demonstrated good agreement of Durnin and Womersly SF equation in estimating body fat of 78 non-obese and obese Caucasian older heathy adults [11] nonetheless it did not perform Lin’s concordance correlation coefficient or Bland and Altman’s analyses as we did. Hence, it is likely that the individuals with higher body fat percentage have high amount of internal fat that were not detected by SF measurements, which could have led to the underestimation by SF equation [31]. Due to the explanations above the SF equation is not accurate enough to be used in clinical settings in older adults with more than 40% of body fat.

The Bland and Altman approach [28] used in our analyses to estimate the average difference and concordance limits between two BF methods has also been used in previous studies [37], wherein it was stated that the use of correlation coefficients may not be appropriate, as a high correlation may not reflect a high level of concordance [28, 37]. The average difference observed in our findings between the methods used and the reference standard indicated that some participants might exhibit considerable diagnostic errors when using either method, since the %BF may be underestimated or overestimated depending on the method used.

In Brazil, the Ministry of Health considers an older adult those people aged 60 years and older while in North America and Europe an older adult is someone aged 65 years and older. This could have been a limitation of this study; however, the mean age of our participants was over 70 years and, therefore, this fact will not limit the comparison of our results with other studies. One possible limitation that could also be stated relates to the generalizability of the findings, due to our sample not being nationally representative. Another potential limitation of our study could be attributed to the fact that the SF assessment is very difficult especially in older adults. However, in order to minimize such errors, we adopted the following strategies: use of good quality skinfold caliper, all anthropometrics were extensively trained in volunteer older adults, measures were taken three times and we provided previous orientation about hydration status.

## Conclusions

BIA and SF equation showed a strong level of concordance to estimate body fat percentage in all participants and among women when compared to our standard reference i.e. DXA. However, both methods underestimated body fat percentage in women and men with high body fat percentage. A strong level of concordance was observed between DXA and the anthropometric equation developed by Durnin and Womersley in men, while BIA had a moderate concordance in this group. Future research in older adults should consider various methods, different ethnic groups and the development of new SF equations.

## Data Availability

The datasets used and/or analysed during the current study are available from the corresponding author on request.
